# Multi-responsive paper chemosensors based on mesoporous silica nanospheres for quantitative sensing of heavy metals in water†

**DOI:** 10.1039/d3ra00369h

**Published:** 2023-02-23

**Authors:** Islam M. El-Sewify, Ahmed Radwan, Hassan Mohamed El-Said Azzazy

**Affiliations:** a Department of Chemistry, Faculty of Science, Ain Shams University 11566 Abbassia Cairo Egypt; b Department of Chemistry, School of Sciences & Engineering, The American University in Cairo SSE, Rm #1194, P.O. Box 74 New Cairo 11835 Egypt hazzazy@aucegypt.edu; c Department of Nanobiophotonics, Leibniz Institute for Photonic Technology Albert Einstein Str. 9 Jena 07745 Germany

## Abstract

Exposure to low concentrations of heavy metal cations seriously harms living organisms, hence they are considered environmental toxins. Portable simple detection systems are required for field monitoring of multiple metal ions. In this report, paper-based chemosensors (PBCs) were prepared by adsorbing 1-(pyridin-2-yl diazenyl) naphthalen-2-ol (chromophore), which recognizes heavy metals, onto filter papers coated with mesoporous silica nano spheres (MSNs). The high density of the chromophore probe on the surface of PBCs resulted in ultra-sensitive optical detection of heavy metal ions and short response time. The concentration of metal ions was determined using digital image-based colorimetric analysis (DICA) and compared to spectrophotometry under optimal sensing conditions. The PBCs exhibited stability and short recovery times. The detection limits determined using DICA of Cd^2+^, Co^2+^, Ni^2+^ and Fe^3+^ were 0.22, 0.28, 0.44, and 0.54 μM; respectively. Additionally, the linear ranges for monitoring Cd^2+^, Co^2+^, Ni^2+^ and Fe^3+^ were 0.44–4.4, 0.16–4.2, 0.8–8.5, and 0.002–5.2 μM; respectively. The developed chemosensors showed high stability, selectivity, and sensitivity for sensing of Cd^2+^, Co^2+^, Ni^2+^ and Fe^3+^ in water under optimum conditions and hold potential for low cost, onsite sensing of toxic metals in water.

## Abstract 2

Paper based chemosensors (PBCs) are prepared by coating mesoporous silica nanospheres on filter papers then immobilizing the probe. PBCs generate different colors in the presence of Co^2+^, Cd^2+^, Ni^2+^, or Fe^3+^ and results are quantified by DICA.

## Graphical abstract

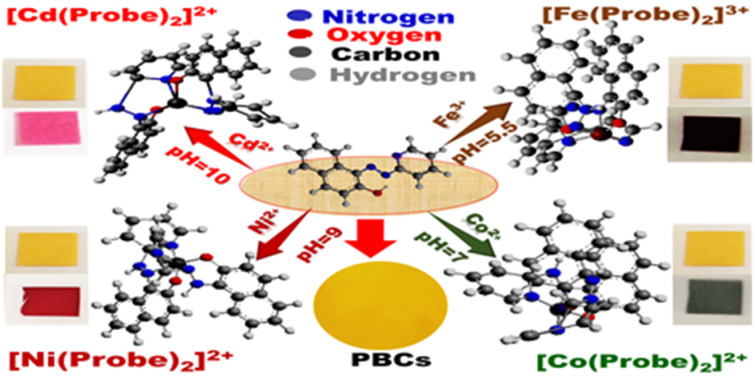

## Introduction

**Scheme 1:**
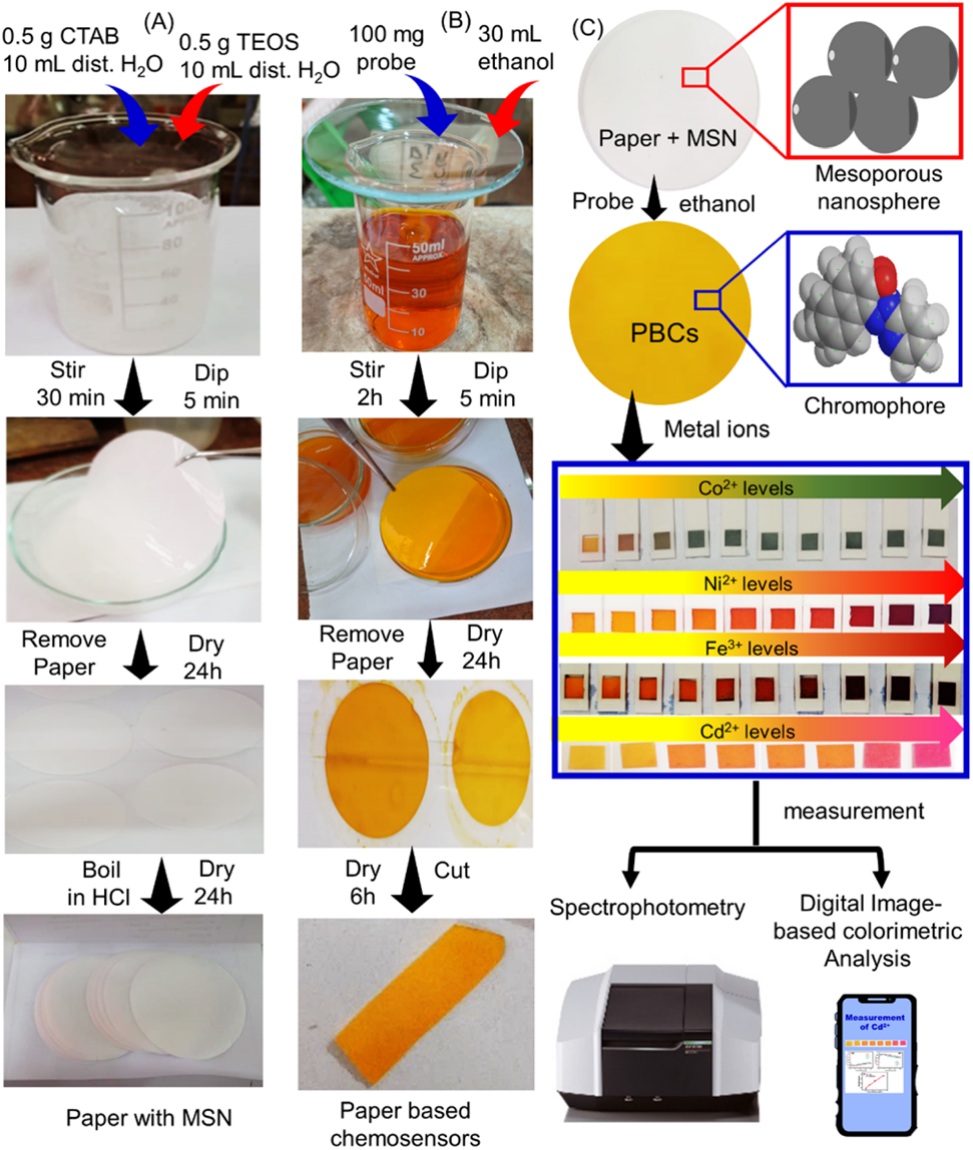
Fabrication of paper based chemosensors (PBCs) for sensing of Ni^2+^, Fe^3+^, Co^2+^ and Cd^2+^. (A) Mesoporous silica nanospheres (MSNs) were loaded over filter paper. (B) The papers with MSN dipped in ethanolic solution of 1-(pyridin-2-yl diazenyl) naphthalen-2-ol (organic chromophore) to prepare PBCs. (C) The PBCs generate different colours upon detection of Ni^2+^, Fe^3+^, Co^2+^ and Cd^2+^ in water under optimum conditions Spectrophotometry and digital image-based colorimetric analysis (DICA) were used for quantification of results.

Heavy metal ions leak into surface or underground water from industrial activities or geological sources and pose a severe risk to living organisms.^1^ Cadmium is widely used in different industries including metal alloys, electroplating, stains, fertilizers, and rechargeable batteries.^8^ Accumulation of cadmium in tissues has been correlated to the enlargement of vital organs, bone weakening, and impairment of the cardiovascular, immunological, and reproductive systems.^9^ Cobalt is used as a catalyst for chemical industries and manufacturing rechargeable lithium-ion batteries.^10^ Although cobalt plays an important function in mammalian metabolism as a trace element; exposure to high cobalt levels has been linked to cardiomyopathy and thyroid hyperplasia.^11–14^ Iron is an essential component of key proteins involved in major physiological functions such as respiration and energy metabolism.^2^ Iron overload, however, leads to damage of vital organs such as liver, heart, and endocrine glands. Nickel is widely used in making Ni–Cd batteries and electroplating.^3^ Exposure to high levels of nickel has been linked to cardiovascular, kidney, and lung diseases.^4–7^ Multiple guidelines for acceptable levels of heavy metals in drinking water were published by different regulatory bodies including the US Environmental Protection Agency, the European Union, and the World Health Organization (Table 1).^15–19^ Various analytical techniques have been established for detection of heavy metal ions such as ICP-AES, ICP-MS, chemiluminescence, atomic absorption spectrometry,^20,21^ solid-phase^22,23^ and fluorescence spectroscopy.^24^ However, they have several drawbacks such as long turnaround time, complicated procedures, need for sophisticated infrastructure, and high cost. Therefore, the challenges for sensing different heavy metals using chromogenic materials and portable chemosensors were investigated in different assays.^25–33^ In this report, multi-responsive PBCs were prepared for monitoring of Cd^2+^, Co^2+^, Fe^3+^, and Ni^2+^ in water. A chromophore probe was immobilized onto mesoporous silica nanosphers (MSNs) for selective detection of various heavy metal ions.^34,35^ MSNs offer several advantages, including large pore volume, high surface area, and homogeneous mesopore size distribution. To generate quantitative results, color generated by the chemosensors upon detection of heavy metals was quantified by digital image colorimetric analysis (DICA) and compared to UV-vis spectroscopy (Scheme 1).^36^ The prepared PBCs exhibited high selectivity, stability, and sensitivity for sensing Cd^2+^, Co^2+^, Fe^3+^, and Ni^2+^ in water under optimum sensing conditions.

**Table 1:** Drinking water quality guidelines (μg L^−1^) for heavy metals

Metal	WHO	EU	US EPA	Oxidation states	Health effects
Cadmium	3	5	5	II	Cardiovascular issues, osteoporosis, cancer.^15^
Cobalt	—	—	100	II, III	Cardiovascular and pulmonary issues.^16^
Iron	—	200	300	II, III	Haemochromatosis, gastrointestinal issues.^17,18^
Nickel	70	20	—	II	Dermatitis, kidney failure^19,20^

## Results and discussion

### Morphology of PBCs

**Fig. 1:**
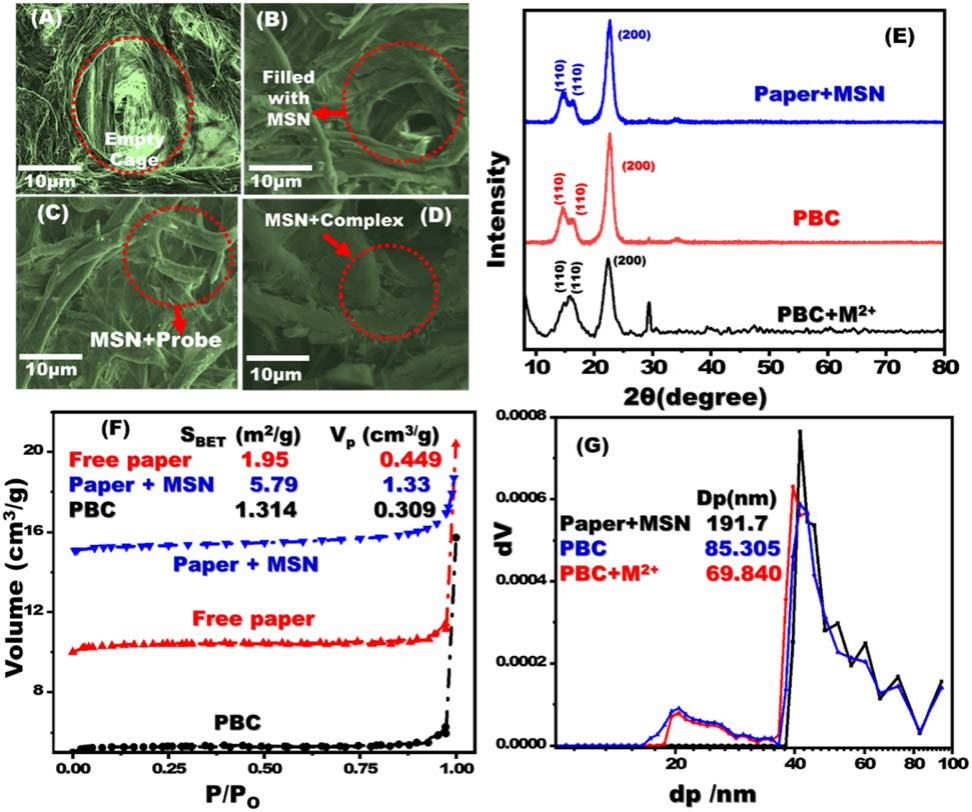
FE-SEM images of free filter paper (A); paper containing mesoporous silica nanospheres (MSNs) (B); MSN coated filter papers after addition of the chromophore probe (C); and interaction of MSNs/carrier/chromophore with metal ion (D). X-ray diffraction patterns of paper containing MSNs, PBCs (MSNs plus chromophore), and complex of PBC with metal ion (E). N_2_ isotherms (F) and NLDFT (G) of paper with MSNs, PBCs and chromophore with metal ion.

Field emission scanning electron microscopy (FESEM) was used to examine the morphology of the filter paper and the filter paper coated with MSNs (Fig. 1A and B). A thin layer of MSN was observed which covered the pores of the cellulose paper. Cellulose fibre cages were blocked after decoration with chromophore (Fig. 1C and D). SEM-EDS analysis and mapping of treated filter paper with MSNs, paper based chemosensors (PBCs), and PBCs + M^2+^ were investigated. The existing of Si element was confirmed the treatment of filter paper with MSNs (Fig. S1A†). The distribution of chromophore elements and metal ions over treated filter paper with MSNs confirms the successful immobilization and complexation respectively (Fig. S1B and C†). The X-ray diffraction patterns of paper with MSNs and PBCs were analyzed (Fig. 1E). The filter paper containing MSNs, PBCs, and PBCs after detection of metal ions revealed specific diffraction peaks ascribed to (110), (200), (110) and (004) planes. The cellulose structure was identified by the resulting XRD data.^37^ The diffraction intensities of the paper containing MSNs reduced significantly after chromophore decoration. Results suggest hydrogen bond interactions between the cellulose chains with MSNs and the chromophore.^38^ FTIR spectroscopy was conducted to investigate the decoration of chromophore into filter paper (Fig. S2†). The existence of band at 797.97, 1053.78, which are referred to siloxane bond (797.97, Si–O–Si bending (1053.78 cm^−1^). Our result confirms the successful grafting of MSNs. The stretching of the –OH group is responsible for the wide band between 3400 and 3200 cm^−1^, whereas the C–H stretching of methylene groups is responsible for the band about 2903 cm^−1^.

The N_2_ isotherms and NLDFT experiments were conducted to determine the pore size and surface area of the fabricated PBCs and their complexes (Fig. 1).^39–41^ Untreated filter paper, paper containing MSNs, optical probe, and complex showed the same isotherm type (III). The surface area of paper containing MSN was 5.79 m^2^ g^−1^ and that of untreated paper was 1.95 m^2^ g^−1^, indicating that MSNs were successfully adsorbed to the filter paper. The PBC surface area decreased dramatically (1.31 m^2^ g^−1^) when they were in combination with metal ions (0.91 m^2^ g^−1^), which suggests the efficient loading of chromophore on the treated filter paper. The NLDFT investigations revealed the presence of different pore sizes in the filter paper containing MSN (Fig. 1G). After the immobilization procedure, the pore diameter of the treated filter paper was dramatically decreased.

### Determination of optimum pH for sensing metal ions

**Fig. 2:**
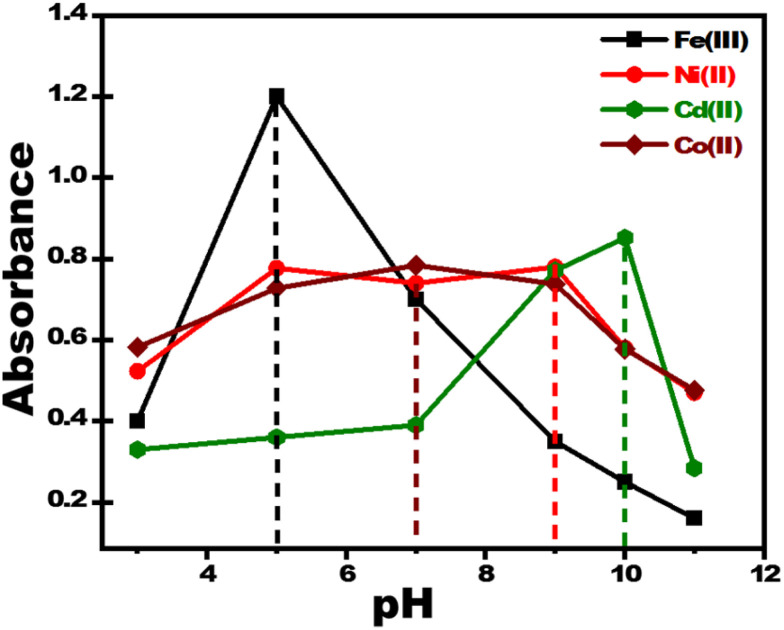
Effect of pH on response of paper-based chemosensors for detection of 2 ppm of Fe^3+^, Co^2+^, Ni^2+^, and Cd^2+^. The optimum pH values for sensing Fe^3+^, Co^2+^, Ni^2+^, and Cd^2+^ were 5.5, 7.0, 9.0 and 10; respectively.

Changes in pH dramatically modify color intensity and dispersion at ultra-trace metal ion concentrations. Standard solutions of metal ions were prepared using buffer solutions at different pH values and the UV-vis absorbance was measured to identify the optimal pH level for metal ion sensing using PBCs. The determined optimal pH values for sensing Fe^3+^, Co^2+^, Ni^2+^and Cd^2+^ were 5.5, 7.0, 9.0 and 10; respectively (Fig. 2).

### Quantitative measurement of metal ions using PBCs and spectrophotometry

**Fig. 3:**
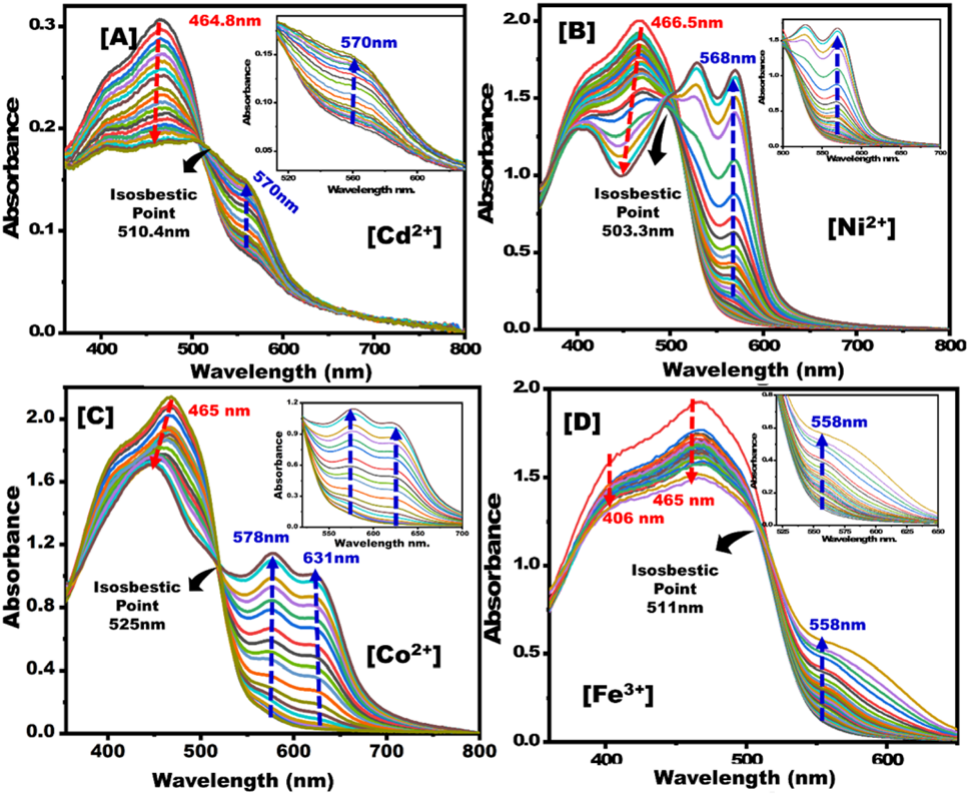
Absorption spectra of paper-based chemosensors upon titration with (A) Cd^2+^, (B) Ni^2+^, (C) Co^2+^, and (D) Fe^3+^ under optimum sensing parameters.

UV-vis absorption spectra were obtained for PBCs treated using increasing concentrations of Co^2+^, Cd^2+^, Ni^2+^, and Fe^3+^. Then the detection ranges (*D*_R_) of each metal ion sensing technique were determined (Fig. 3). PBCs kits offered one-step detecting methods for both qualitative and quantitative determination of Co^2+^, Cd^2+^, Ni^2+^, and Fe^3+^.

**Fig. 4:**
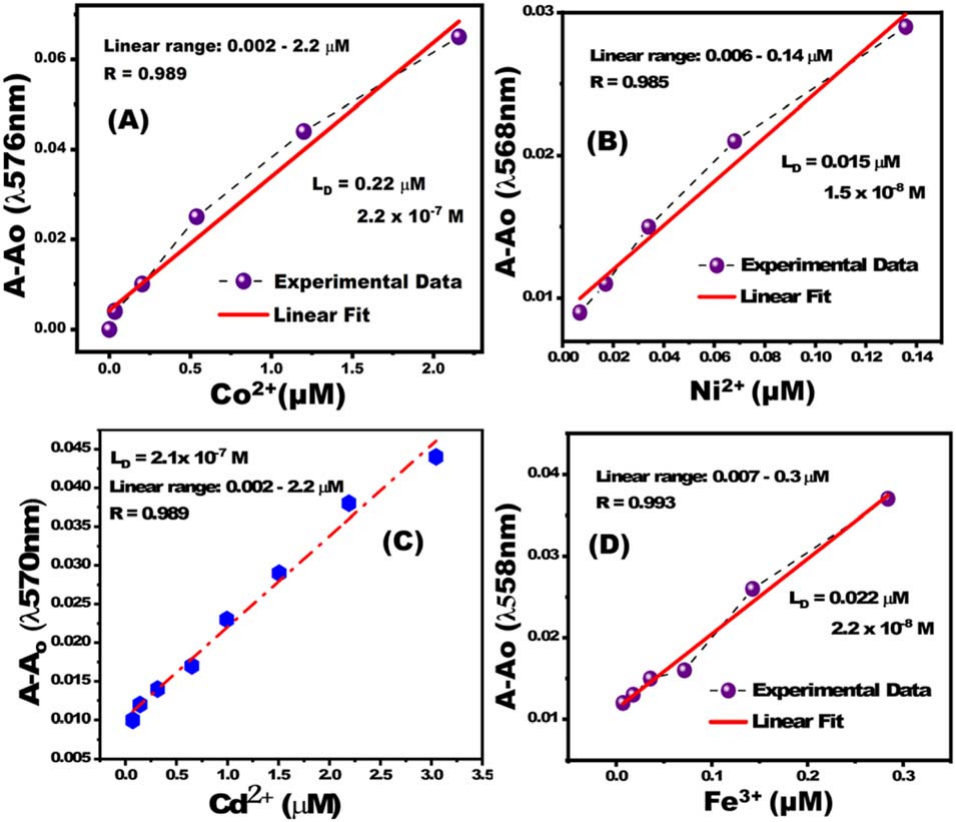
Calibration curves for paper-based chemosensors for (A) Co^2+^ at *λ*_570nm_, (B) Ni^2+^ at *λ*_568nm_, (C) Cd^2+^ at *λ*_570nm_, and (D) Fe^3+^ at *λ*_558nm_.

The calibration curves of PBCs were linear at low concentration ranges of Co^2+^, Cd^2+^, Ni^2+^, and Fe^3+^ (Fig. 4). The determined limits of detection (*L*_D_) suggest that the fabricated PBCs have recognized ultra low concentrations of target ions, as compared to sensors fabricated by conventional strategyies. PBCs based on MSNs enabled, for the first time, efficient metal ion detection down to ∼10^−9^ mol L^−1^ (Table 2). The fabricated PBCs exhibited greater recognition of Co^2+^, Cd^2+^, Ni^2+^, and Fe^3+^ compared to other chemosensors that have been described previously (Table 3).

**Table 2:** Analytical parameters for detection of metal ions using paper-based chemosensors and spectrophotometric analysis^a^

Metal ion	*L*_D_ (μM)	*D*_R_ (μM)	*R*_t_ (s)	Optimum pH
Co^+2^	0.22	0.002–2.2	20	7
Cd^+2^	0.13	0.002–8.8	10	10
Ni^+2^	0.015	0.006–0.14	15	9
Fe^+3^	0.022	0.007–0.3	15	5.5

a Limit of detection (*L*_D_), detection range (*D*_R_), response time (Rs).

**Table 3:** Analytical parameters for detection of metal ions using paper-based chemosensors and digital image-based colorimetric analysis

Detection principle	Sensor	Metal Ion	*L*_D_ (μM)	Ref.
Absorbance	Cubic mesocage sensors	Cd^2+^	0.0307	42
Absorbance	Cd^2+^ chemosensors	Cd^2+^	0.19	43
Absorbance	Dithizone TiO_2_ sensor	Cd^2+^	0.0156	44
Absorbance	Aluminosilica optical sensor	Cd^2+^	0.0024	45
		Co^2+^	0.0028	
Absorbance	Green AuNP probe	Cd^2+^	0.03	46
Absorbance	Functionalized AuNP	Cd^2+^	0.0629	47
Absorbance	Azo-HNTA probe	Co^2+^	0.77	48
Absorbance	CpAD probe	Co^2+^	0.0066	49
Absorbance	Chemosensor for cobalt	Co^2+^	1.8	50
Absorbance	Chemosensor (HL) based on coumarin	Co^2+^	0.31	51
Fluorescence	Eu(iii)-organic framework	Fe^3+^	23	52
Fluorescence	Uranyl organic framework	Fe^3+^	0.0992	53
Fluorescence	Eu^3+^post-functionalized UiO-66	Fe^3+^	12.8	54
Colorimetric	Multicomponent sensor	Fe^3+^	1.26	55
Fluorescence	Aryl hydrazones of β-diketones	Ni^2+^	7	56
Absorbance	Alizarin complexone	Ni^2+^	40	57
Fluorescence	Hydrazine carbothioamide	Ni^2+^	0.079	58
Absorbance	Quinoline derivative	Ni^2+^	0.22	59
Absorbance	Chalcone based ratiometric chemosensor	Ni^2+^	5.14	60
Absorbance	Paper based chemosensors (PBCs) using spectrophotometry	Cd^2+^	0.13	This work
		Co^2+^	0.22	
		Ni^2+^	0.015	
		Fe^3+^	0.022	
Absorbance	Paper based chemosensors (PBCs) using digital image-based colorimetric analysis (DICA)	Cd^2+^	0.22	This work
		Co^2+^	0.28	
		Ni^2+^	0.44	
		Fe^3+^	0.54	

### Quantitative determination of metal ions using PBCs and digital image-based colorimetric analysis (DICA)

**Fig. 5:**
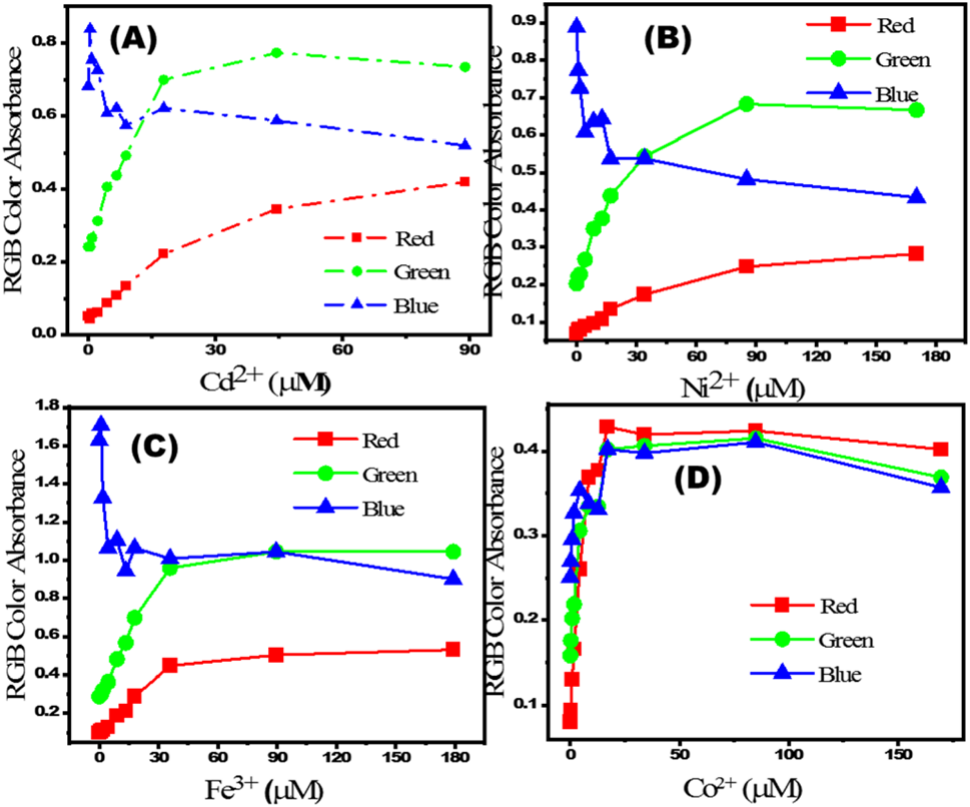
Relationship between (A) [Cd^2+^], (B) [Ni^2+^], (C) [Fe^3+^] and (D) [Co^2+^] and their calculated absorbances from RGB of images captured using a mobile camera.

**Fig. 6:**
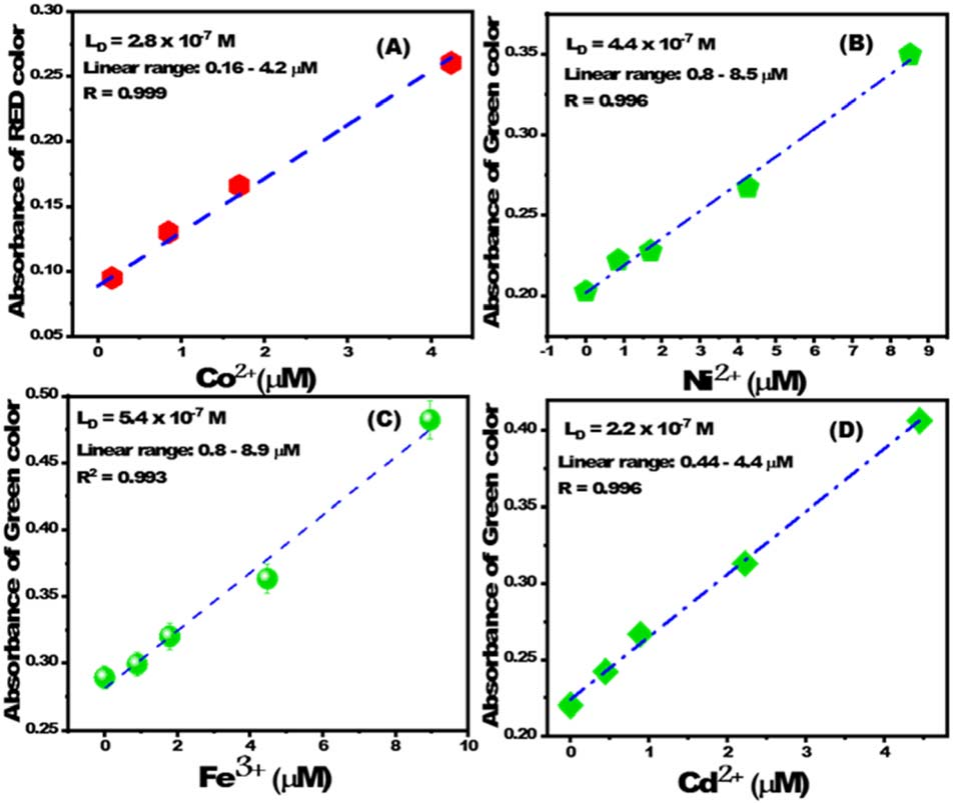
The linear correlation between absorbance of (A) red color and [Co^2+^], (B) green color and [Ni^2+^], (C) green color and [Fe^3+^], and (D) green color and [Cd^2+^].

The change in RGB intensity values (IR, IG, and IB) of PBCs were examined using the images of PBC colors (obtained using cell phone camera) which developed upon sensing different concentrations of Co^2+^, Cd^2+^, Ni^2+^, and Fe^3+^ in water. The obtained images of PBCs kits were analysed using Adobe Photoshop CS6. Scheme 1C shows the digital images of PBCs with different concentrations of Co^2+^, Cd^2+^, Ni^2+^, and Fe^3+^. The mean integer value for each RGB component decreased when an intense color was created as the metal ion concentration increased (Table 4). As shown in Fig. 5, the relationships between color absorbance and the concentrations of Co^2+^, Cd^2+^, Ni^2+^, and Fe^3+^ agreed with those obtained by spectrophotometry (Fig. 3). The Co^2+^, Cd^2+^, Ni^2+^, and Fe^3+^ concentrations and the absorbance of the mean integer value for each RGB component were shown to be linearly correlated, demonstrating that these ions may be detected with great sensitivity at extremely low concentrations (Fig. 6). The *L*_D_ and LQ of metal ions using DICA are in agreement with those obtained by spectrophotometry. Therefore, DICA can be utilized as a low cost, portable, and semi-quantitative analytical method for sensing Co^2+^, Cd^2+^, Ni^2+^, and Fe^3+^ recognized by the developed PBCs.

**Table 4:** Recognition of metal ions using PBCs and digital image-based colorimetric analysis

Metal ion	*L*_D_ (mol L^−1^)	*D*_R_ (mol L^−1^)	*R*_t_ (s)	Specific pH
Co(ii)	2.8 × 10^−7^ (16.5 ppb)	(0.16–4.2) × 10^−6^	20	7
Cd(ii)	3.1 × 10^−7^ (34.85 ppb)	(0.44–4.4) × 10^−6^	10	10
Ni(ii)	4.4 × 10^−7^ (25.824 ppb)	(0.8–8.5) × 10^−6^	20	9
Fe(ii)	5.4 × 10^−7^ (30.15 ppb)	(0.002–5.2) × 10^−6^	20	5

### Selectivity analyses

**Fig. 7:**
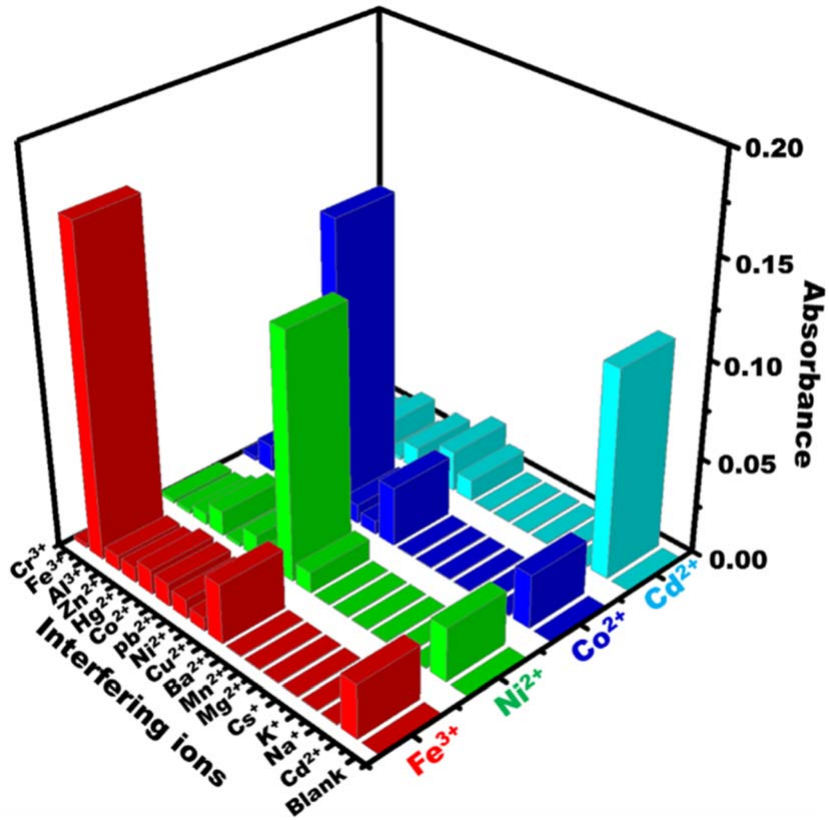
Effect of common interfering cations on absorbance spectra of paper-based chemosensors under optimum sensing conditions (Co^2+^ at *λ*_570nm_, Ni^2+^ at *λ*_568nm_, Cd^2+^ at *λ*_570nm_, and Fe^3+^ at *λ*_558nm)_.

The selectivity of PBCs towards Cd^2+^, Co^2+^, Ni^2+^, and Fe^3+^ was investigated under optimal sensing conditions. Known concentrations of possible interferring cations were added to 0.5 ppm of Co^2+^, Cd^2+^, Ni^2+^, and Fe^3+^ and solutions tested using the PBCs at the specific ion sensing pH values of 5.5, 7, 9, and 10 for Fe^3+^, Co^2+^, Ni^2^ and Cd^2+^; respectively. For each target ion, and in the presence of interfering ions, there were no significant variations in the PBC absorption spectra or visual color patterns as detection of metal ions was carried out at their specific pH values (Fig. 7). Table S1† illustrates the tolerance concentrations of several interfering ions.

### Proposed sensing mechanism of PBCs

**Scheme 2:**
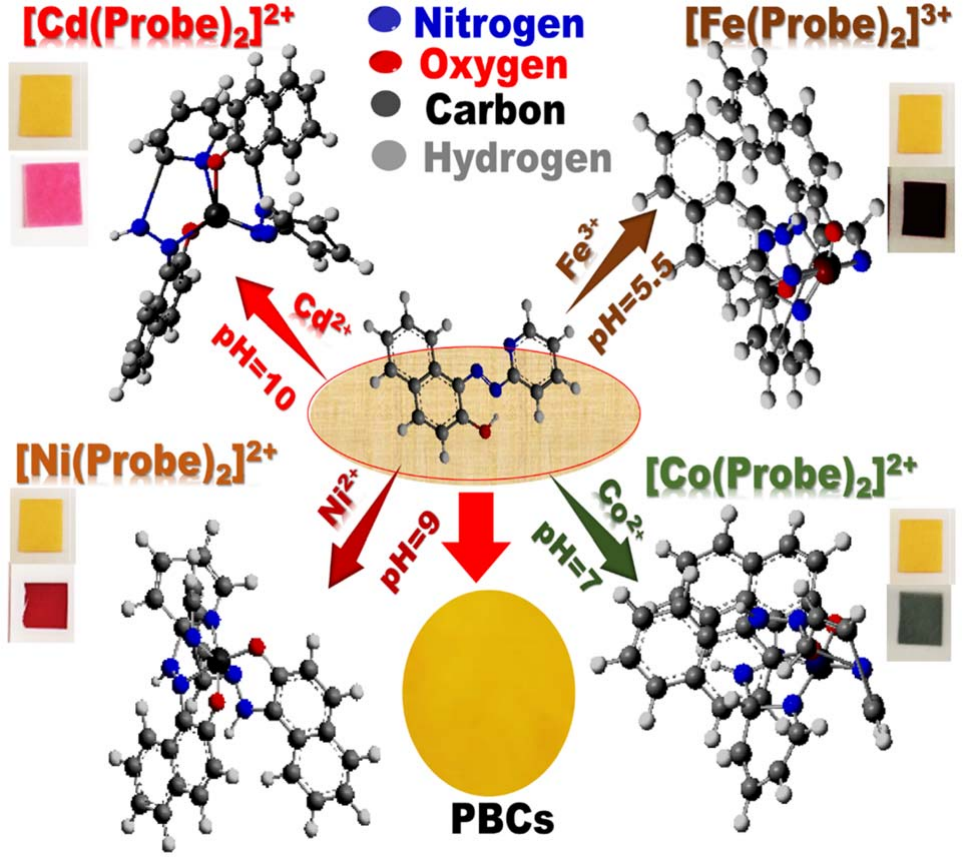
. Schematic diagram of the possible interactions of the paper-based chemosensors (PBCs) with Cd^2+^, Co^2+^, Fe^3+^ and Ni^2+^ under optimal sensing conditions. Upon the formation of different complexes between the optical probe and the heteroatom, the color of PBCs changes: (a) from yellow to pink in presence of Cd^2+^ at pH = 10; from yellow to dark green in presence of Co^2+^ at pH = 7, (c) from yellow to red in presence of Ni^2+^ at pH = 9, and (d) from yellow to dark brown in presence of Fe^3+^ at pH = 5.5.

Under optimum sensing conditions, commercial filter papers treated with MSNs then covered with the optical probe were employed to detect Co^2+^, Cd^2+^, Ni^2+^, and Fe^3+^ (Scheme 2). When oxygen and azo-nitrogen of organic probe on PBCs are available for complexation with Co^2+^, Cd^2+^, Ni^2+^, or Fe^3+^, stable complex with two coordination spheres are formed. As indicated in Scheme 2, the stoichiometric ratio of Co^2+^, Cd^2+^, Ni^2+^, and Fe^3+^ to organic probe at a specific pH is predicted to be 1 : 2. The results show that raising Co^2+^, Cd^2+^, Ni^2+^, and Fe^3+^ concentrations improved absorption spectra (Fig. 3). Furthermore, complex formation and charge transfer were linked to the occurrence of an isosbestic point. The developed PBCs enable naked-eye detection of ultra-trace metal ion concentrations without the need for complicated traditional techniques.

### Using PBCs for detection of metal ions in water

PBCs were used to test several drinking water samples to identify Co^2+^, Cd^2+^, Ni^2+^, and Fe^3+^. Water samples were collected from a variety of places and spiked with known concentrations of Co^2+^, Cd^2+^, Ni^2+^, and Fe^3^. Under optimum sensing conditions, PBCs were dipped into the water samples and different colors developed immediately in presence of different metal ions (Table S2†). Water samples were tested three times for Co^2+^, Cd^2+^, Ni^2+^, and Fe^3+^, then the results were quantified using DICA. Results obtained from DICA analysis were concordant to those obtained by spectrophotometry (Table S2†). Elution studies were carried out using various concentrations of EDTA to determine the best eluent for adsorbent renewal and reusability (Fig. S3†). The designed optical chemosensors for sensing Fe(iii), Co(ii), Ni(ii), and Cd(ii) ions can be used multiple times.

To the best of our knowledge, this study is the first to develop multi-responsive optical chemosensors based on mesoporous silica that incorporates simplicity but still retains sensitivity, selectivity, and fast-response detection of target ions in drinking and environmental water samples. Coating the filter papers with mesoporous silica nanospheres as carriers of the probe improved the sensing functionality for detection of ultra-trace concentrations of metal ions in water samples. The optical chemosensors have uniform structural morphology enabled by utilizing mesoporous silica nanospheres as carriers for sensing the ultra-trace concentration of metal ions in water resources. The detection limit of sensing ultra-trace metal ions is 100 times lower than other reported method. The developed optical chemosensors can be regenerated, using 0.1 M EDTA solution, and reused multiple times. Superior selectivity of optical chemosensors for their target cations under optimal sensing conditions (pH, *etc*) even in the presence of multiple interfering cations. Colorimetric techniques can be used to measure color intensities of the chemosensors and generate quantitative results. The fabricated optical chemosensors exhibited high stability (in water and on shelf).

Clearly, this study supports several of the United Nations Sustainability Development Goals including good health and well-being, clean water and sanitation, and industry and innovation. The designed paper based chemosensors (kits) are of low cost compared to similar commercial ones which would enable large scale accessibility by populations in low- and middle-income countries. It is also of note that the produced chemosensors can be regenerated and used multiple times. Additionally, similar chemosensors can be optimized for detection of toxic metals in cosmetics. Finally, future studies can investigate the use of the developed strategy for removal of heavy metals from water.

## Experimental

### Chemicals

All experiments were conducted using Milli-Q water. CoCl_2_, NiCl_2_, FeCl_3_, CdCl_2_, diethyl ether, CH_3_COONa, CH_3_COOH, 1-(pyridin-2-yl diazenyl) naphthalen-2-ol (C_15_H_11_N_3_O), tetraethyl orthosilicate (TEOS), acetone, and cetyltrimethyl ammonium bromide (CTAB), were obtained from Sigma-Aldrich (St. Louis, MO). NaOH, and Na_2_HPO4 were acquired from El-Nasr Pharmaceuticals (Cairo, Egypt).

### Fabrication of PBCs

MSNs were prepared at pH = 7 and applied on filter papers as described previously with minor modifications.^28^ CTAB (0.5 g) was mixed with 100 mL of distilled water for 30 min. Ethanol (10 mL) and TEOS (2.5 mL) were then added and the solution mixed for another 0.5 h (Scheme 1A). Then, 1.5 mL of NaOH was added and the solution stirred for 2 h. The filter papers were immersed in the prepared MSN solution multiple times (5 min each). The papers containing MSN were dried at 50 °C for 6 h then dipped in ethanolic solution (30 mL) of 1-(pyridin-2-yl diazenyl) naphthalen-2-ol (100 mg) for multiple times (5 min each) as shown in (Scheme 1B). To produce the PBCs which were then cut into appropriate size (1 cm^2^) and placed into 3D printed holders (Scheme 1C).

### Analysis of toxic metal ions

Metal ion solutions (200 ppm) were prepared in Milli-Q water. The PBC was placed in a quartz cuvette then a known concentration of a specific metal ion was added and sonicated for 5 s. UV-vis spectroscopic spectra were measured within seconds (without shaking). Colorimetric detection of different concentrations of Cd^2+^, Co^2+^, Fe^3+^, and Ni^2+^ using PBCs were performed at different pH. UV-vis spectra were obtained and after equilibration, a prominent color change was observed and the PBC absorbance spectra obtained. The color change of PBCs was also quantified using DICA.

## Conclusions

PBCs were fabricated by adsorbing mesoporous silica nanosphere carrier, prepared using a low-cost method, on filter papers then decorated with the optical chromophore 1-(pyridin-2-yl diazenyl) naphthalen-2-ol. Optimal sensing conditions for each PBC were determined. The MSN-based PBCs enabled visual detection of Co^2+^, Cd^2+^, Ni^2+^, and Fe^3+^ present in water with high sensitivity and selectivity. The PBCs allowed detection of multiple ions and featured long-term stability and could be utilized several times using a simple regeneration process. Quantitative results were obtained by analysis of images (obtained using cell phone camera) of colored PBCs using DICA which were similar to those obtained with spectrophotometry. This possibly is the first report that employs PBCs based on MSNs for detecting multiple heavy metal ions in water using DICA or UV-vis spectroscopy. Additional PBCs could be developed using similar strategies and optimized for detection of additional metals in water and other matrices.

## Author contributions

Conceptualization, I. M. E, H. M. E. A.; methodology and data collection, A. R. and I. M. E.; writing, reviewing and editing, all authors; project administration and funding, H. M. E. A. All authors have written and reviewed the final version of the manuscript.

## Conflicts of interest

HMEA is an inventor on a granted patent on developing optical chemosensors for detection of toxic metals.

## Supplementary Material

RA-013-D3RA00369H-s001
