# Supplementary material for: Multi-responsive paper chemosensors based on mesoporous silica nanospheres for quantitative sensing of heavy metals in water
Source: RSC Adv. 2023 Feb 23;13(10):6433–41. doi: 10.1039/d3ra00369h (PMC9947744; doi:10.1039/d3ra00369h)
Supplement: RA-013-D3RA00369H-s001 [file RA-013-D3RA00369H-s001.pdf]

Electronic Supplementary Material (ESI) for RSC Advances.
This journal is © The Royal Society of Chemistry 2023

# Supporting information

## Multi-Responsive Functionalized Mesoporous Silica Paper Based Chemosensors for Quantitative Sensing of Heavy Metals in Water

Islam M. El-Sewify [1,2], Ahmed Radwan [1,2] , Hassan M. E. Azzazy [2,3*]

1 Department of Chemistry, Faculty of Science, Ain Shams University, Abbassia, Cairo 11566, Egypt

[2]Department of Chemistry, School of Sciences & Engineering, The American University in Cairo, SSE, Rm #1194, P.O. Box 74, New Cairo 11835, Egypt

[3]Department of Nanobiophotonics, Leibniz Institute for Photonic Technology, Jena 07745, Germany

*Corresponding author

Email: hazzazy@aucegypt.edu

**Instruments**

The surface properties including the specific surface area and the pore structure were determined by $N_2$ adsorption-desorption isotherms which were measured by automatic surface area and pore size analyzer (BELSORP MINI X, Microtrac MRB) was used to determine Specific surface area ($S_{BET}$) via adsorption-desorption of N2 gas at 77 K via applying the Barrett–Joyner–Halenda (BJH) method. The pore size distribution was determined from the adsorption isotherms by using nonlocal density functional theory. $S_{BET}$ was calculated using multi-point adsorption data from linear segment of the $N_2$ adsorption isotherms using Brunauer-Emmett-Teller (BET) theory. Before the $N_2$ isothermal analysis, all samples were pre-treated at 80°C for 6 h and calibrated to $10^{-3}$ Torr. Small- and wide-angle powder X-ray diffraction (SAXRD, and, respectively) patterns were measured by using X'Pert - PRO – TAM with monochromated CuKα (λ = 1.54060 Å) radiation with diffraction reflections recorded for 2 angles between 5◦ and 10◦ corresponding to d- and WAXRD of chemosensors scanned from 4° to 80°. Prior to analysis, the samples were outgassed at 80°C for 24 h. The Field emission scanning electron microscopy (FESEM) images were used to investigate the morphologies and obtained by Zeiss Leo Supra55 microscope. Moreover, to record the SEM micrographs, the scanning electron microscope was operated at 20 keV. The samples for FESEM observations were examined after copper coating. The scanning

electron microscope was operated at 20 keV. The absorbance spectra of the PBCs chemosensors were measured by a Shimadzu UV-2600 solid-state UV–vis spectrophotometer.

**Apparatus and Software**.

Scanning of the color scales was performed using the scanner of LaserJet Pro 400 in color mode RGB 24 bits with resolution 300 dpi and Digital Nikon camera (D3100). The determination of the colorimetric characteristics of the obtained images and transformation of the color coordinates were performed in the Adobe Photoshop CC 2017 (64 Bit) on a personal laptop. The crop tool called "Elliptical Marquee Tool" was used for selecting an oval region in the middle of the image of the colored sample and the ''Histogram" tool used to obtain the average color intensities values of Red, Green, and Blue (RGB) of each image. All data were transferred to Microsoft Excel 2016 (64 Bit) spreadsheet for subsequent data analysis and using Origin Pro 2016 (64 Bit) for data plotting. The color intensity can be also directly related to the absorbance concentration using the following equation:

$$A_X = -log\frac{(I_X - I_{X,b})}{(I_{X.w} - I_{X,b})} = -log\frac{(I_X)_c}{(I_{X,w})_c} = -\log R_X$$

where for each color X: (R, B, G), $A_X$ is the absorbance of X, $I_X$ and $R_X$ are the intensity and the reflectance of light X, respectively, $I_{X,b} = 0$, $I_{X,W} = 256$, and C is the concentration of X.
The limit of detection (LOD) was calculated using the following equation (3.3XSD of intercept/slope). The Origin lab software was used to determine the standard deviation of the intercept of the drawn calibration curve.

**Table S1.** Tolerance concentration for interfering matrix species during recognition of [0.5 ppm] Fe(III), Co(II), Ni(II) and Cd(II) ions by using the PBCs.

| **Analytes** | **Specific pH** | **Tolerance limits for foreign cations (ppm)** | | | | | | | | | |
|---|---|---|---|---|---|---|---|---|---|---|---|
| | | **Na(I)** | **$Cu^{2+}$** | **$Cd^{2+}$** | **$Co^{2+}$** | **$Ni^{2+}$** | **$Pb^{2+}$** | **$Hg^{2+}$** | **$Zn^{2+}$** | **$Al^{3+}$** | **$Fe^{3+}$** |
| **Co(II)** | 7 | 100 | 5 | 5 | Ref | 5 | 100 | 100 | 100 | 100 | 10 |
| **Cd(II)** | 10 | 100 | 5 | Ref | 5 | 5 | 100 | 100 | 100 | 100 | 10 |
| **Ni(II)** | 9 | 100 | 5 | 5 | 5 | Ref | 100 | 100 | 100 | 100 | 10 |
| **Fe(III)** | 5 | 100 | 5 | 5 | 5 | 5 | 100 | 100 | 100 | 100 | Ref |

## Table S2. Determination of $Co^{2+}$, $Cd^{2+}$, $Ni^{2+}$, and $Fe^{3+}$ using PBCs in water samples.

| Sample | ICP-MS Analysis (ppm) | Metal Ions | Amount spiked µg/L ($x10^{-3}$ppm) | Proposed approach PBCs | | |
|---|---|---|---|---|---|---|
| | | | | Recovery ±RSD % µg/L ( $x10^{-3}$ppm) | E% | Error% |
| Sample 1 | 0.155 ppm $Al^{3+}$, 0.06 ppm $Ba^{2+}$, 0.0034 ppm $Bi^{3+}$, 524.9 ppm $Ca^{2+}$, 0.0007 ppm $Cd^{2+}$, 0.0025 ppm $Co^{2+}$, 0.0082 ppm $Cu^{2+}$, 1.73 ppm $Fe^{2+}$, 1.76 ppm $Fe^{3+}$, 0.0002 ppm $Hg^{2+}$, 0.14 ppm $Li^{+}$, 1066 ppm $Mg^{2+}$, 1.05 ppm $Mn^{2+}$, 0.0088 ppm $Mo^{4+}$, 0.0124 ppm $Ni^{2+}$, 0.0044 ppm $Pb^{2+}$, 0.95 ppm $Si^{2+}$, 20.3 ppm $Sr^{3+}$, 0.0006 ppm $Tl^{4+}$, 0.0005 ppm $V^{5+}$, 0.147 ppm $Zn^{2+}$ | $Co^{2+}$ | 20 | 20.54±1.1 | 98.8 | 2.7 |
| | | | 50 | 50.13±0.48 | 98.3 | 0.26 |
| | | | 100 | 98.06±0.85 | 98.5 | 1.94 |
| Sample 2 | 0.034 ppm $Al^{3+}$, 0.035 ppm $Ba^{2+}$, 616.9 ppm $Ca^{2+}$, 0.0005 ppm $Cd^{2+}$, 0.0008 ppm $Co^{2+}$, 0.014 ppm $Cr^{6+}$, 0.11 ppm $Fe^{2+}$, 0.08 ppm $Fe^{3+}$, 0.0005 ppm $Hg^{2+}$, 0.064 ppm $Li^{+}$, 301.3 ppm $Mg^{2+}$, 0.0056 ppm $Mn^{2+}$, 0.0076 ppm $Mo^{4+}$, 0.0017 ppm $Ni^{2+}$, 0.0072 ppm $Pb^{2+}$, 11.21 ppm $Si^{2+}$, 33.43 ppm $Sr^{3+}$, 0.0192 ppm $Zn^{2+}$ | $Cd^{2+}$ | 10 | 9.62 ±0.52 | 96.2 | 3.8 |
| | | | 50 | 47.9 ±0.73 | 95.8 | 4.2 |
| | | | 100 | 96.7 ±1.3 | 96.7 | 3.3 |
| Sample 3 | 0.0144 ppm $Al^{3+}$, 0.086 ppm $Ba^{2+}$, 77.22 ppm $Ca^{2+}$, 0.0008 ppm $Cd^{2+}$, 0.0007 ppm $Co^{2+}$, 0.0127 ppm $Cr^{6+}$, 0.0094 ppm $Cu^{2+}$, 0.173 ppm $Fe^{2+}$, 0.12 ppm $Fe^{3+}$, 0.0008 ppm $Hg^{2+}$, 0.0219 ppm $Li^{+}$, 32.04 ppm $Mg^{2+}$, 0.0039 ppm $Mn^{2+}$, 0.0068 ppm $Mo^{4+}$, 0.017 ppm $Ni^{2+}$, 44.51 ppm $Si^{2+}$, 4.26 ppm $Sr^{3+}$, 0.0019 ppm $Tl^{4+}$, 0.0072 ppm $V^{5+}$, 0.0035 ppm $Zn^{2+}$ | $Ni^{2+}$ | 10 | 9.91 ±0.41 | 99.1 | 0.9 |
| | | | 50 | 49.4±0.49 | 98.8 | 1.2 |
| | | | 100 | 99.1 ±0.72 | 99.1 | 0.9 |
| Sample 4 | 0.0468 ppm $Al^{3+}$, 0.02 ppm $Ba^{2+}$, 480.9 ppm $Ca^{2+}$, 0.0004 ppm $Cd^{2+}$, 0.0001 ppm $Co^{2+}$, 0.13 ppm $Fe^{2+}$, 0.14 ppm $Fe^{3+}$, 0.0007 ppm $Hg^{2+}$, 0.082 ppm $Li^{+}$, 180.1 ppm $Mg^{2+}$, 0.0037 ppm $Mn^{2+}$, 0.0092 ppm $Mo^{4+}$, 0.0014 ppm $Ni^{2+}$, 0.0124 ppm $Pb^{2+}$, 17.8 ppm $Si^{2+}$, 17.86 ppm $Sr^{3+}$, 0.005 ppm $Zn^{2+}$ | $Fe^{3+}$ | 10 | 9.78 ±0.38 | 97.8 | 2.2 |
| | | | 50 | 49.2 ±1.05 | 98.4 | 1.6 |
| | | | 100 | 98.6 ±1.17 | 98.6 | 1.4 |

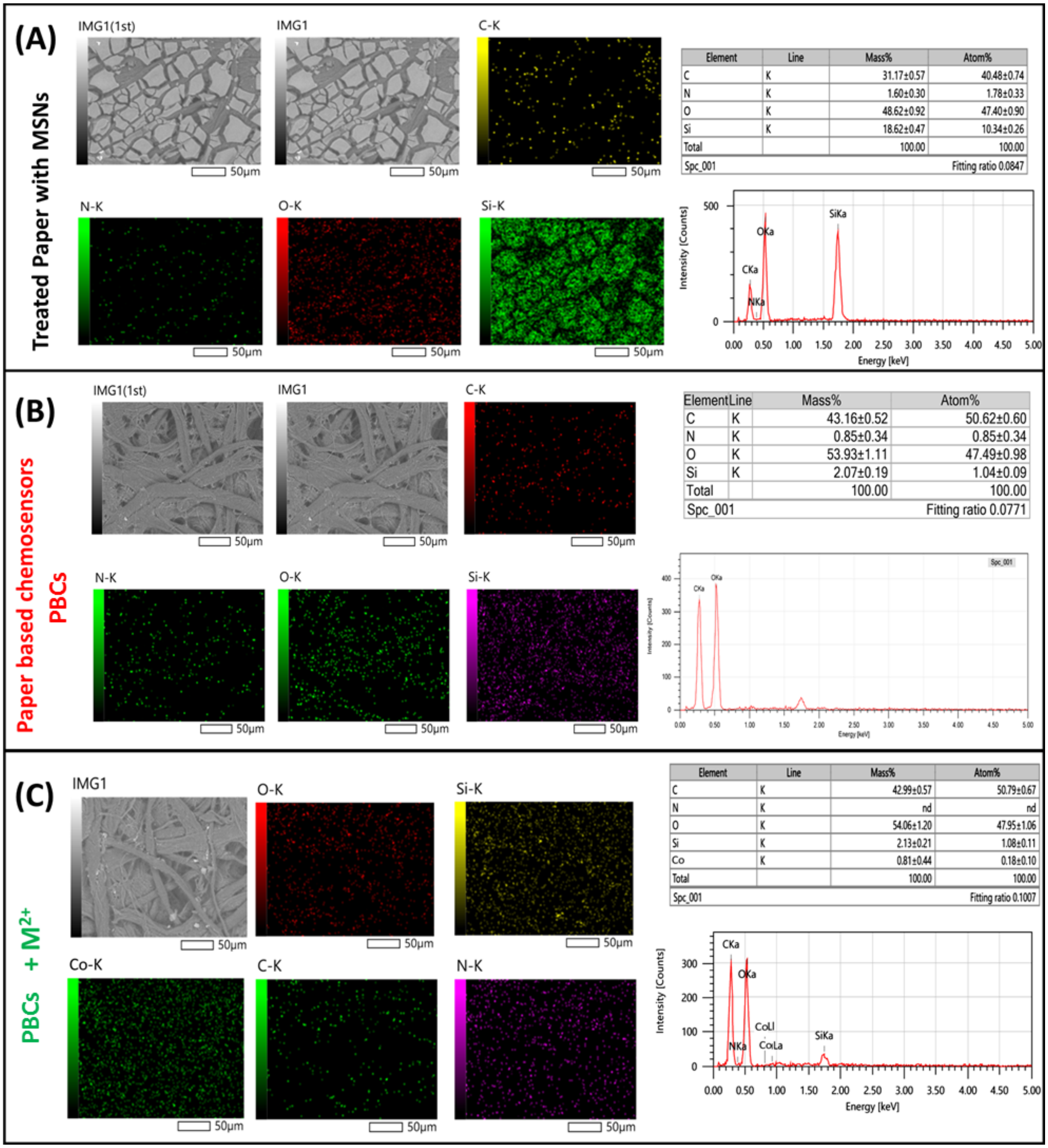


**Figure S1**. SEM-EDS analysis and mapping of (A) treated filter paper with mesoporous silica nanospheres, (B) paper based chemosensors (PBCs), and (C) PBCs with $Co^{2+}$.

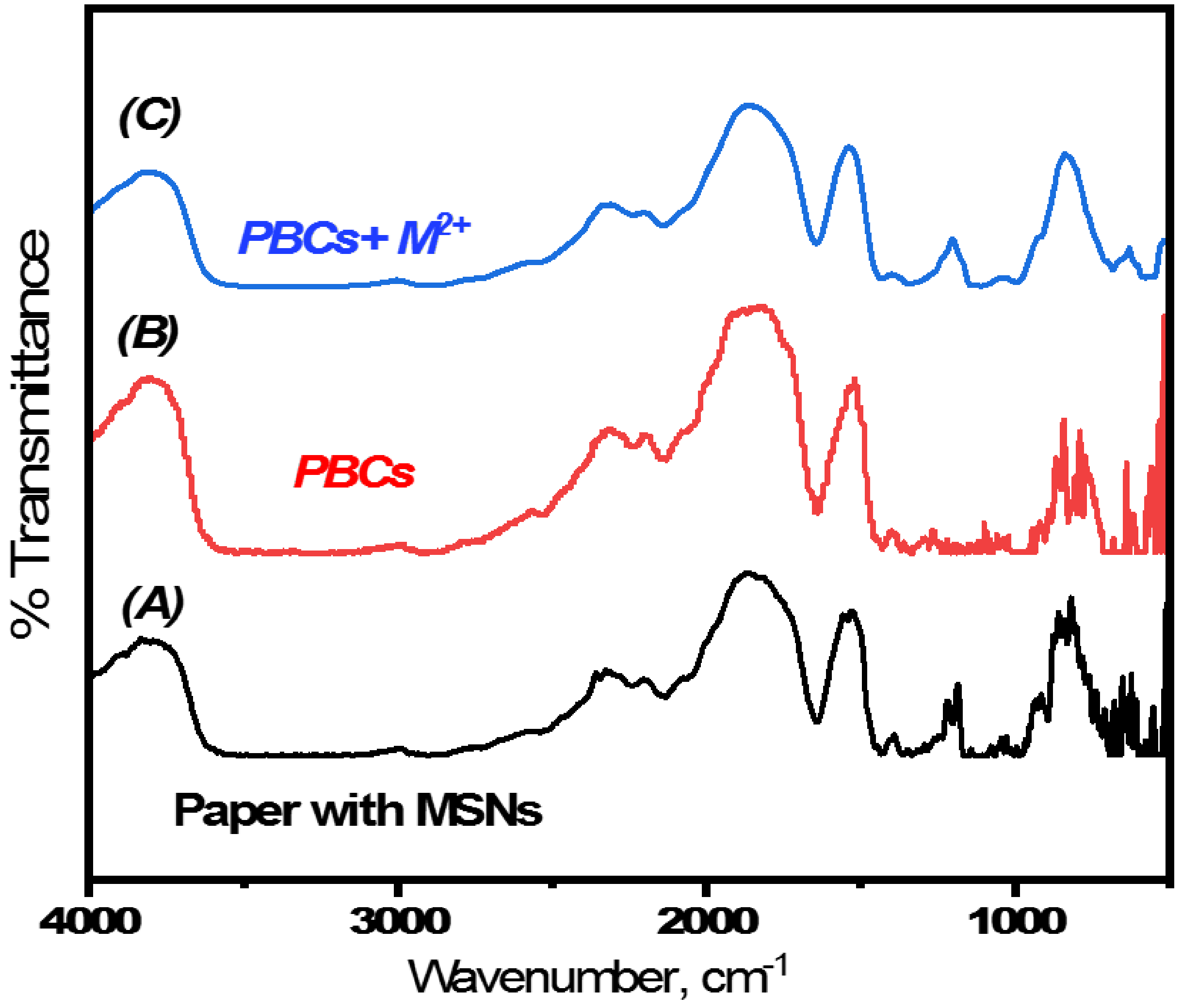


**Figure S2.** FTIR spectra of (A) filter paper treated with mesoporous silica nanospheres (MSNs), (B) paper based chemosensors (PBCs), and (C) PBCs with $M^{2+}$.

**Regeneration and Reuse of PBCs**

**Metal ion recovery via elution/desorption is an essential metric to consider when evaluating materials with regard to cost-effectiveness. Elution studies were carried out using various concentrations of EDTA to determine the best eluent for adsorbent renewal and reusability. A simple treatment with 0.1 M EDTA was shown to efficiently remove Fe(III), Co(II), Ni(II), and Cd(II) ions (i.e. decomplexation) while preserving the adsorbent's residual functionality for reuse in several cycles. To liberate the metal ions and get a "metal–free" sensor surface, these procedures were repeated numerous times using a liquid-exchange method. After being regenerated and cleaned with water, the optical chemosensors were vacuum dried before being reused. PBCs were regenerated/reused for five cycles. Figure S3 shows that increasing the recovery cycles had a minor effect on the sensitivity of the produced optical**

**chemosensors. Accordingly, the constructed optical chemosensors for sensing Fe(III), Co(II), Ni(II), and Cd(II) ions can be used multiple times.**

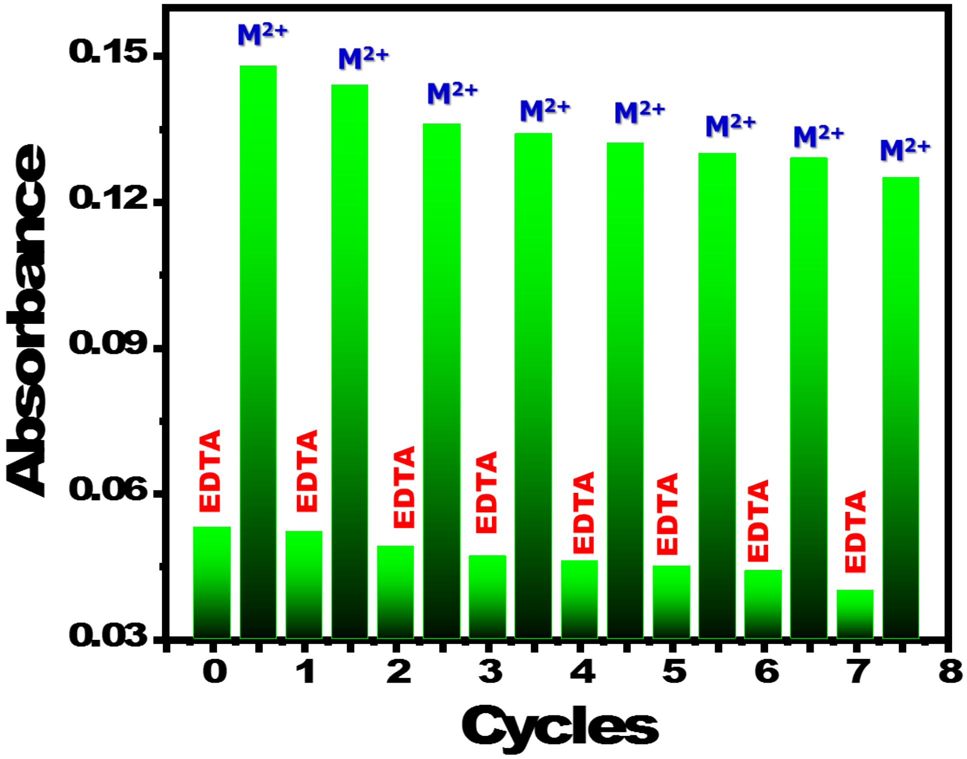


Figure S3. Elution and regeneration of PBCs for multiple cycles using 0.1 M EDTA as the stripping agent.
